# “Extravasation injuries requiring plastic surgery - a retrospective single center study”

**DOI:** 10.1016/j.jpra.2026.02.003

**Published:** 2026-02-08

**Authors:** Moritz Rudolf Milewski, Martynas Tamulevicius, Anieto Onochie Matthias Enechukwu, Florian Bucher, Frederik Julius Kloss, Frederik Schlottmann, Louisa Jutta Dietz, Nico Franke, Luis Alberto Barros Navarro, Lisa Lorbeer, Peter Maria Vogt

**Affiliations:** Department of Plastic, Aesthetic, Hand and Reconstructive Surgery, Hannover Medical School, Carl Neuberg-Straße 1, 30625 Hannover, Germany

**Keywords:** Extravasation, Surgery, Treatment, Chemotherapy, Subcutaneous wash out

## Abstract

**Background:**

Extravasation of intravenous agents can lead to severe tissue injury, including necrosis and compartment syndrome, sometimes necessitating complex surgical intervention. Identifying factors that predict poor outcomes is crucial for guiding management. This study aimed to determine predictors of severe complications in patients with extravasation injuries referred for plastic surgery consultation.

**Methods:**

We conducted a retrospective, single-center analysis of 37 patients who required non-conservative management for extravasation injuries between 2014 and 2024. The primary outcome was the development of a any complication that requires surgical treatment like skin necrosis or compartment syndrome. A Bayesian logistic regression model was employed to identify predictive factors, including the use of a subcutaneous wash-out procedure, catheter type, and agent type (vesicant vs. irritant vs. not harmful).

**Results:**

The subcutaneous wash-out procedure was strongly associated with a reduced likelihood of severe complications. All other independent variables like days from incident to referral to plastic surgery, Volume administered and type of extravasated agent were not significant.

**Conclusion:**

In this cohort of severe extravasation injuries requiring surgical consultation, the use of subcutaneous wash-out was associated with fewer severe complications accountable to its protective effect as well as correct patient selection as subcutaneous wash-out is not suitable for the treatment of necrosis or infection.

## Introduction

Extravasation of intravenous agents is a significant iatrogenic complication characterized by the inadvertent leakage of infused fluids or medications from the vascular compartment into the surrounding tissue.^1^ While infiltration refers to the leakage of non-vesicant solutions, extravasation specifically denotes the escape of potentially damaging or vesicant substances, which can result in a spectrum of local tissue injury. This event most commonly arises when the integrity of the vein is compromised—whether due to mechanical factors such as catheter dislodgement, vein fragility, or improper cannulation technique, or patient-related factors such as extremes of age, pre-existing vascular pathology, or impaired communication ability.^2^

Extravasation of intravenous agents result in widely varying outcomes. The severity of injury is determined by several factors: the physicochemical properties of the extravasated agent (such as cytotoxicity, osmolality, and pH), the volume and duration of tissue exposure, and the anatomical site of the event.^2^ Certain medications, notably chemotherapeutic agents and vasoactive drugs, are particularly notorious for causing irreversible tissue damage upon extravasation.^3^ Studies of chemotherapeutic and antitumor agents describe a spectrum of immediate tissue injury - from mild erythema to severe ulceration and necrosis.^4^ Here, the severity of local tissue damage from an extravasation primarily depends on the specific drug involved. These agents are categorized into three types based on their potential to cause harm (as outlined in Table 1)^5^, ^6^, ^7^, ^8^, ^9^, ^10^:

**Table 1:** Chemotherapeutic agents divided into three groups according to their toxicity upon extravasation.

Vesicans	Irritans	Non vesicans
Amsacrin	Bendamustin	(Peg)-Asparaginase
Brentuximab vedotin^a^	Busulfan	5-FU^c^
Cabazitaxel	Carboplatin	Aflibercept
Cisplatin > 0.4 mg/mL	Cyclophosphamid	Arsentrioxid
Dactinomycin	Dacarbazin	Azacytidin
Daunorubicin	Doxorubicin liposomal	Bleomycin
Docetaxel	Etoposid	Bortezomib
Doxorubicin	Fotemustin	Cetuximab
Epirubicin	Gemcitabin	Etoposid phosphat
Idarubicin	Ifosfamid	Fludarabin
Mitomycin C	Irinotecan	Methotrexat
Mitoxantron	Melphalan	Pemetrexed
Nab-paclitaxel	Daunorubicin liposomal	Pentostatin
Oxaliplatin^b^	Temozolomid	Rituximab
Paclitaxel	Teniposid	Thiotepa
Vinblastin	Trastuzumab-Emtansin	Topotecan
Vincristin	Treosulfan	Trastuzumab

*Note. Adapted from AWMF Guideline*.^26^

a Little or no *experience* with extravasation; classification is based in part on data from the respective manufacturers.

b The assessment of the damage pattern varies depending on the concentration (e.g., bolus administration, higher dilution).

c Very high risk of phlebitis with continuous infusion.

*Vesicants (Tissue-Necrosing Agents)*: These are agents that cause necrosis, with anthracyclines being the most common example. A vesicant can induce blistering and ultimately lead to the destruction of local tissue, resulting in necrosis and ulceration.

*Irritants*: These agents typically cause pain and/or inflammation at the injection site or along the vein, but generally do not cause necrosis. However, the extravasation of a large volume may lead to small ulcers in the surrounding soft tissue.

*Non-Vesicants*: These agents do not typically cause any local tissue reaction upon extravasation. Some, including Bleomycin and Methotrexate, are considered safe enough for intramuscular injection.

In contrast, investigations of vasopressor extravasations report mostly minor local injuries, although one study noted major disability in 4.4% of cases.^3^ Evaluations of non-cytotoxic agents (including intravenous nutrition and calcium) showed mixed outcomes, with some instances reaching severe necrosis.^29^

Conservative management—including measures such as cooling, elevation, site monitoring, and administering antidotes (e.g., dexrazoxane against anthracyclines and hyaluronidase against vinca alkaloids) - yielded high rates of resolution (up to 98% avoiding surgery).^4^ Then, there are minimally invasive procedures like the injection of saline to dilute the possibly cytotoxic agent.^11^, ^12^ If combined with liposuction, it is known as a subcutaneous wash-out procedure (SWOP), first reported in 1993.^13^ Both options are very much capable of alleviating the risk of necrosis and fibrosis.^11^, ^12^^,^^14^ Still, some extravasations require non minimally invasive surgical treatment. These range from immediate incision and drainage due to elevated compartment pressure, to excessive secondary debridement of necrotic tissue and following flap surgery to cover the defect.^15^

Reports note that such extravasations sometimes require delayed surgical management and account for 0.5–6% of chemotherapy‐related adverse events.^16^

Given the potential for significant morbidity, prompt recognition and management of extravasation is essential. Preventive strategies include careful patient and site selection, vigilant monitoring during infusion, and education of healthcare professionals regarding risk factors and early signs of extravasation.

Thus, our objective was to determine predictive factors of complicated extravasation occurrences which required attention from our single center experience in a university department of plastic surgery.

## Methods

### Study design and setting

This study was conducted as a retrospective single-center analysis of extravasation requiring plastic surgery involvement that occurred between January 2014 and April 2024. The study was performed at a major University Hospital and tertiary referral center, which treats approximately 50,000 stationary patients annually. Patient data were collected from hospital records. All patients included in the study provided broad consent for the use of their data in research, and as such, no additional patient consent was required for this specific analysis. Ethics committee approval was waived, as the use of anonymized patient data falls under the institution’s broad consent policy for retrospective studies.

The primary outcome was the occurrence of any serious extravasation-related complication, defined as the presence of necrotic tissue, thrombophlebitis, sensomotoric deficit, compartment syndrome or infection.

### Participants

The study population consisted of individuals who sustained extravasation injuries during the study period. Inclusion criteria were: (1) confirmed diagnosis of extravasation, (2) in-patient non-conservative treatment (surgical management (including subcutaneous wash-out, debridement, or reconstructive surgery) at any point between January 2014 and April 2024 and (3) referral to the Department of Plastic, Aesthetic, Hand and Reconstructive Surgery. Exclusion criteria were: (1) patients managed exclusively with conservative measures (e.g., elevation, cooling, antidotes alone) without surgical intervention and (2) patients whose records were incomplete or whose diagnosis code was incorrect and did not represent a true extravasation injury. There were no other exclusion criteria based on age or gender as pediatric patients were also included in the study and all eligible patients were included in the analysis. The data were collected retrospectively from available medical records.

### Surgical technique

All surgical interventions, including the Subcutaneous Wash-Out Procedure (SWOP), were performed in an operating theater under sterile conditions. SWOP was standardized following a modified Gault technique: multiple small stab incisions were placed circumferentially around the extravasation site, followed by the infiltration of isotonic saline into the subcutaneous space. The volume of infiltrated fluid was titrated based on anatomical location, patient age, skin tension, and surgeon’s discretion. Water jet-assisted liposuction was subsequently performed using a 3-mm blunt cannula to evacuate the diluted agent. To facilitate the continued efflux of residual fluids, the incision edges were only loosely approximated or left open. All procedures were performed by or under the direct supervision of a board-certified plastic surgeon.

### Reporting

All results were reported in accordance with the STROBE guidelines for observational studies.^17^

### Bias

Several inherent limitations and potential biases must be acknowledged in this study. First, the retrospective design at a specialized university hospital introduces a significant selection bias; our cohort represents only severe extravasations requiring plastic surgery consultation while excluding milder cases managed successfully by floor staff. This is compounded by referral bias, as our center treats a patient population with higher systemic morbidity and treatment complexity than found in primary care settings. Furthermore, information bias may exist due to our reliance on historical electronic health records, where documentation granularity regarding exact extravasation volumes or precise timing of interventions can vary over a 10-year period. Finally, as a single-center study, the generalizability of our results should be interpreted within the context of our specific institutional expertise and specialized surgical infrastructure.

### Study size

The study was not designed with a predetermined sample size or power calculation.

### Plotting

Ggplot2^17^ package was used to produce the combined line, dot and bar plots. The package humapr^18^ was used to create the chloropeths of the human body.

### Statistical analysis

The primary analytical goal was to identify predictors associated with the odds of developing the binary outcome variable “Extravasation complications (yes/no)”. Extravasation complications specifically mean those which had to be treated surgically like infection, necrosis or compartment syndrome. Age of the patient and time from incident to referral to plastic surgery were included in the model as a continuous variable, all other variables independent variables were categorial. These are sex, the type of extravasated agent, classified into unknown, non-harmful, irritant, high risk irritant, and vesicant, the type of catheter used for i.v. administration (port, peripheral central) and the usage of SWOP. Because SWOP is a therapeutic intervention performed after the injury to prevent complications, it was included in the model. The volume of extravasated fluid was not included due to high uncertainty of the actual amount due to bleeding, leakage, withdrawal of the catheter and discard of the agent before careful measurement of the administered amount etc. The initial approach was a frequentist generalized linear regression model. However, initial model fitting attempts revealed the issue of almost complete separation in the data. To overcome this methodological challenge, a Bayesian logistic regression model was employed.^19^ A binomial family with a logit link function was specified for the binary outcome. Weakly informative priors—specifically the default normal (location = 0, scale = 2.5, autoscale = TRUE) priors were used to guide the model while still allowing the data to dominate the final estimates. The model’s posterior distribution was estimated using Markov Chain Monte Carlo (MCMC) sampling, running 4 independent chains for 2000 iterations each, with the first 1000 iterations discarded as warmup. Model convergence was confirmed by ensuring the potential scale reduction factor R̂ for all parameters was equal to 1.0. Then, the respective odds ratio was calculated with its corresponding confidence intervals (CIs) that were calculated for the model coefficients to provide a measure of precision. The statistical analysis was conducted using R version 4.4.1 for Mac (R Foundation for Statistical Computing, Vienna, Austria).^20^ The final model was implemented using the rstanarm package.^21^ For descriptive statistics, frequencies and proportions were reported for categorial variables. Continuous variables were summarized using means and interquartile ranges.

## Results

### Demographic data

**Figure 1:**
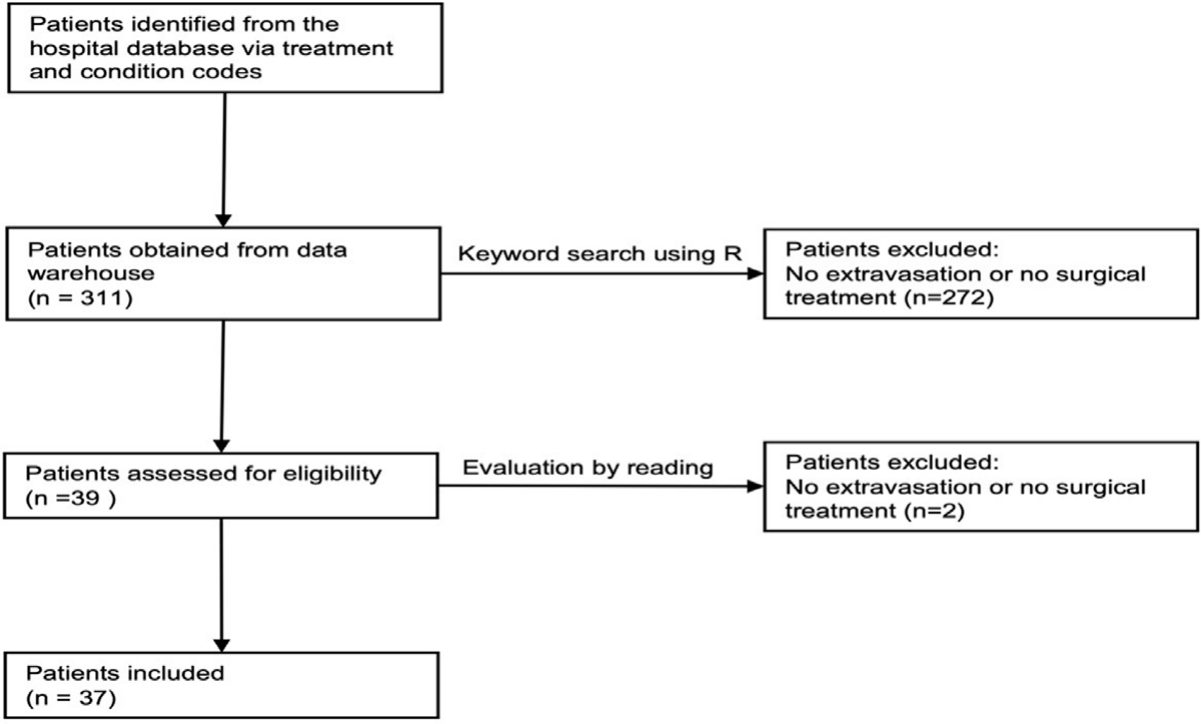
Flow chart for patient exclusion and inclusion.

311 patient were obtained as provided by our internal IT service using an “International Statistical Classification of Diseases and Related Health Problems” code (ICD code) and “Operationen- und Prozedurenschlüsse” (OPS) code search approach. Of these, 274 records were excluded after primary evaluation. Reasons for exclusion included incorrect diagnostic coding, injuries not representing true extravasations, or cases managed entirely conservatively without surgical consultation. A secondary, thorough scrutiny of the remaining records led to the exclusion of two additional patients due to incomplete data, resulting in a final study cohort of 37 patients (Figure 1). There were no missing data other than those already indicated in the demographics table. No specific follow-up period was defined, as patients are typically not referred back to our department after discharge. Follow-up is limited to only a few cases.

Patient demographics are detailed in Table 2, Table 3. Total procedure counts in Table 2 may exceed the number of patients (*n* = 37), as some individuals underwent sequential procedures (e.g., initial debridement followed by secondary flap surgery).

**Table 2:** Patient demographics table.

Category	Absolute count/Median	Frequency/[Q1-Q3]
Sex		
Female	21	56.8%
Male	16	43.2%
Age	63	[47–71]
Age category		
Pediatric (<18)	4	10.8%
Adult (≥18)	33	89.2%
Days from incident to referral	5	[1–16]
Volume of extravasated agent in mL	55	[20–100]
Antidote		
Dexrazoxane	1	1/37
Hyaluronidase	1	1/37
No antidote	35	94.3%
Catheter		
Central venous catheter	1	1/37
Peripheral venous catheter	29	78.4%
Port	7	18.9%
Surgical treatment		
Subcutaneous wash out	15	40.5%
Incision and drainage	4	10.8%
Compartment release	5	13.5%
Debridement	18	48.6%
Skin graft	8	21.6%
Advancement flap	4	10.8%
Transposition flap	1	1/37
Interosseus posterior flap	1	1/37
ALT flap	2	2/37
Complications		
Compartment syndrome	6	16.2%
Infection	6	16.2%
Necrosis	10	27%
Thrombophlebitis	3	3/37
Sensomotoric deficit	1	1/37
Extravasation agent type		
Unknown	2	5.4%
Non-harmful	7	18.9%
Irritant	18	48.6%
High-risk irritant	4	10.8%
Vesicant	6	16.2%
Affected body part		
Foot	2	2/37
Hand	7	18.9%
Forearm	19	51.4%
Arm	1	1/37
Chest	7	18.9%
Neck	1	1/37

*Note.* Percentages are calculated based on the total cohort (*n* = 37). Continuous variables are presented as Median [Interquartile Range, Q1–Q3] unless otherwise specified. For sub-categories with *n* < 4, raw ratios (n/N) are provided in lieu of percentages to avoid the over-interpretation of small denominators. Categories may have cumulative sums exceeding the total patient count, as individual patients may have undergone sequential procedures or presented with multiple clinical complications.

**Table 3:** Root diagnosis related to the administration of the extravasated agent.

Category	Diagnosis	Count	Related extravasation agent
Oncological			
	Breast cancer	3	Epirubicin Epirubicin Paclitaxel
	Lymphoma	3	Contrast agent Doxorubicin/cyclophsphamid Unknown chemotherpy
	Pancreatic cancer	2	folfirinox Nutriflex
	Gastric cancer	1	Nutriflex
	Nasopharyngeal carcinoma	1	Unknown chemotherapy
	Oropharyngeal carcinoma	1	Contrast agent
	Oral squamous cell carcinoma	1	Contrast agent
	Small cell lung cancer	1	Etoposid Cisplatin
	Suspected tumor recurrence	1	Contrast agent
	Urothelial carcinoma	1	Enfortumab
Cardiovascular			
	Atrial fibrillation	1	Amiodaron
	Atrioventricular septal defect	1	Glucose
	Cardiogenic shock	1	Nutriflex
	Endocarditis	1	Rifampicin + Vancomycin
	Suspected heart defect	1	Contrast agent
	Ventricular tachycardia	1	Amiodaron
Neurological			
	Multiple sclerosis	2	Mitoxantron Mitoxantron
	Stroke	2	Contrast agent Contrast agent
	Status epilepticus	1	Propofol
	Traumatic brain injury	1	Nutriflex
Surgical/Trauma			
	Abdominal adhesions	1	Novalgin
	Burn wound	1	Propofol
	Hand amputation	1	Erythrocyte concentrate
	Liver transplantation	1	Bicarbonate
	Thyroidectomy	1	Calcium
Other			
	Iron deficiency anemia	1	Sodium ferric gluconate complex
	Reduced vigilance	1	Glucose
	Gastroscopy	1	Propofol
	Childbirth	2	Sodium ferric gluconate complex Sodium ferric gluconate complex

**Figure 2:**
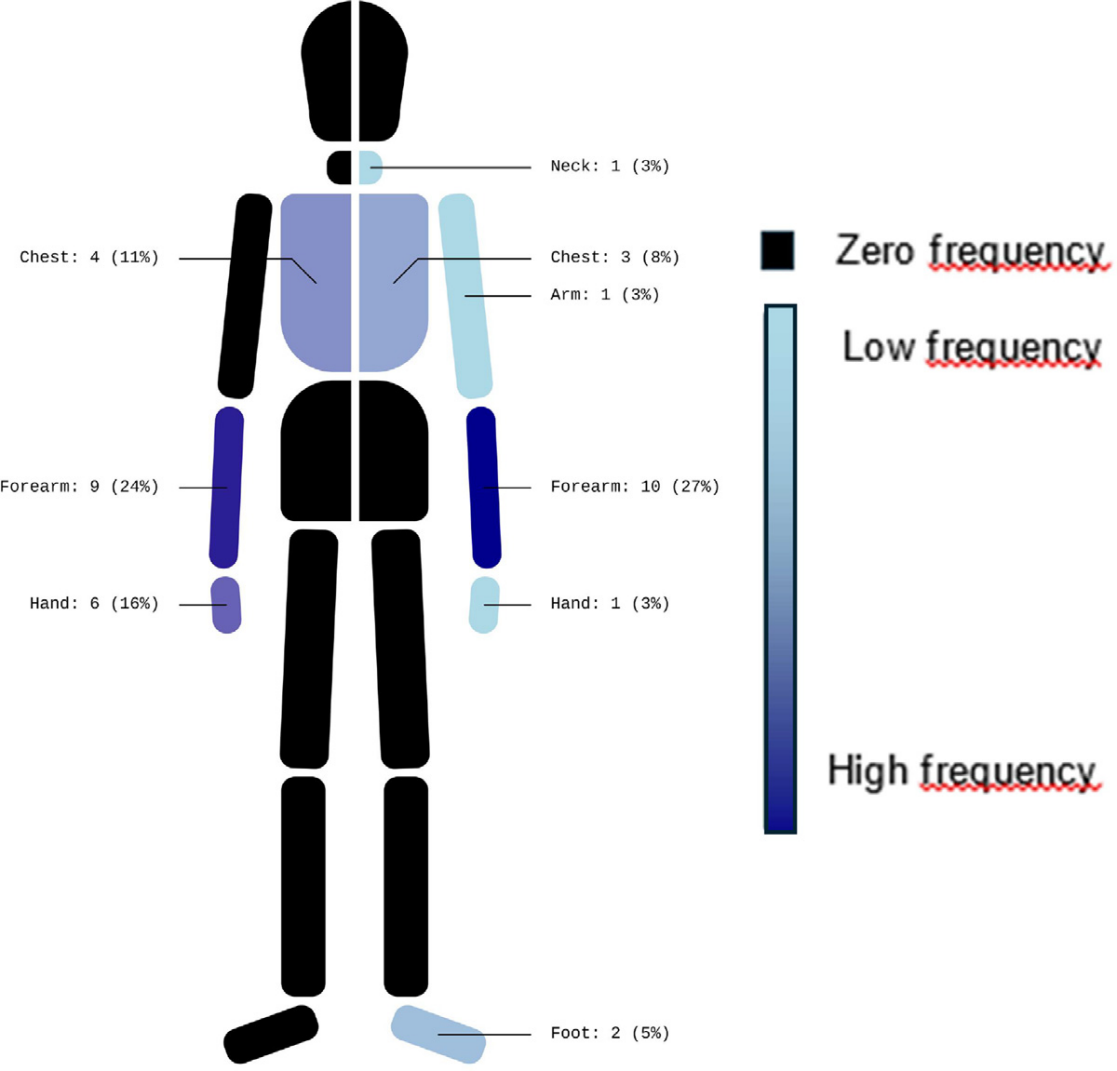
Distribution of Injured Body Parts. Note. This figure illustrates the distribution of extravasation across different body parts, presented as a human plot. Each body section is color-coded from light to dark blue to indicate the frequency of extravasation, with darker blue representing higher frequencies. Black corresponds with zero counts. Absolute numbers of extravasation locations are displayed for each body part, followed by the corresponding frequency in parentheses. The color gradient reflects the frequency of extravasation and does not indicate the severity of the injuries. The most frequently affected areas were the hand and forearm.

Our data indicate an even distribution of extravasation injuries between sexes. The majority of extravasation occurred on the hand and forearm. Affected body parts mimicked the type of intravenous catheter as the majority of extravasation stemmed from peripheral venous catheters in the forearm and hand. The minority of 4 on the right and 3 on the left chest were due to the improper puncture of a subcutaneous port and only one central venous catheter (CVC) was the cause for an extravasation in the neck (Figure 2). The median time from injury to plastic surgical referral was 5 days (IQR: 1–16 days). The administered volume of extravasated agent was 55 in median (IQR: 22–100 days).

### Exemplary case with follow-up

Of all the patients receiving surgical treatment of an extravasation injury, only two received an anterolateral thigh flap to cover a skin defect. One of these is presented below. This patient, a middle-aged man, had sustained an extravasation injury from an unknown chemotherapeutic agent following diffuse large cell lymphoma treatment to the fossa cubiti. Initial evaluation and debridement were performed at another hospital a 4 days after the incident. Subsequently, the patient was transferred to our facility for further treatment. This patient represents a classic delayed intervention and referral. The initial chemotherapeutic extravasation was managed conservatively at an external facility for 4 days. No initial SWOP or saline dilution was performed. By the time of plastic surgical consultation (Day 5), the patient presented with fixed flexion contracture and established necrosis of the cubital fossa. This highlights the “window of opportunity” for SWOP; because the agent was not evacuated early, deep fibrosis occurred, necessitating radical debridement and the subsequent ALT flap.

**Figure 3:**
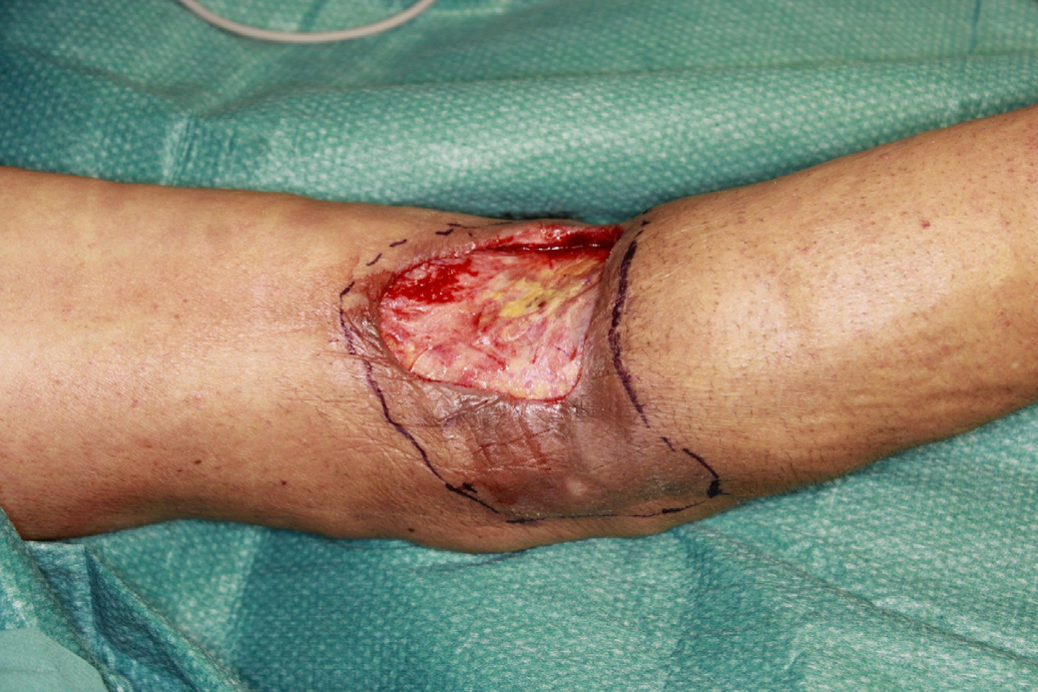
Exemplary extravasation wound of the right elbow at first assessment.

Upon admission, the patient was transferred to the operating room to begin to assess the wound (Figure 3) and debrided fibrotic and necrotic tissue. Note the partial debridement performed externally.

**Figure 4:**
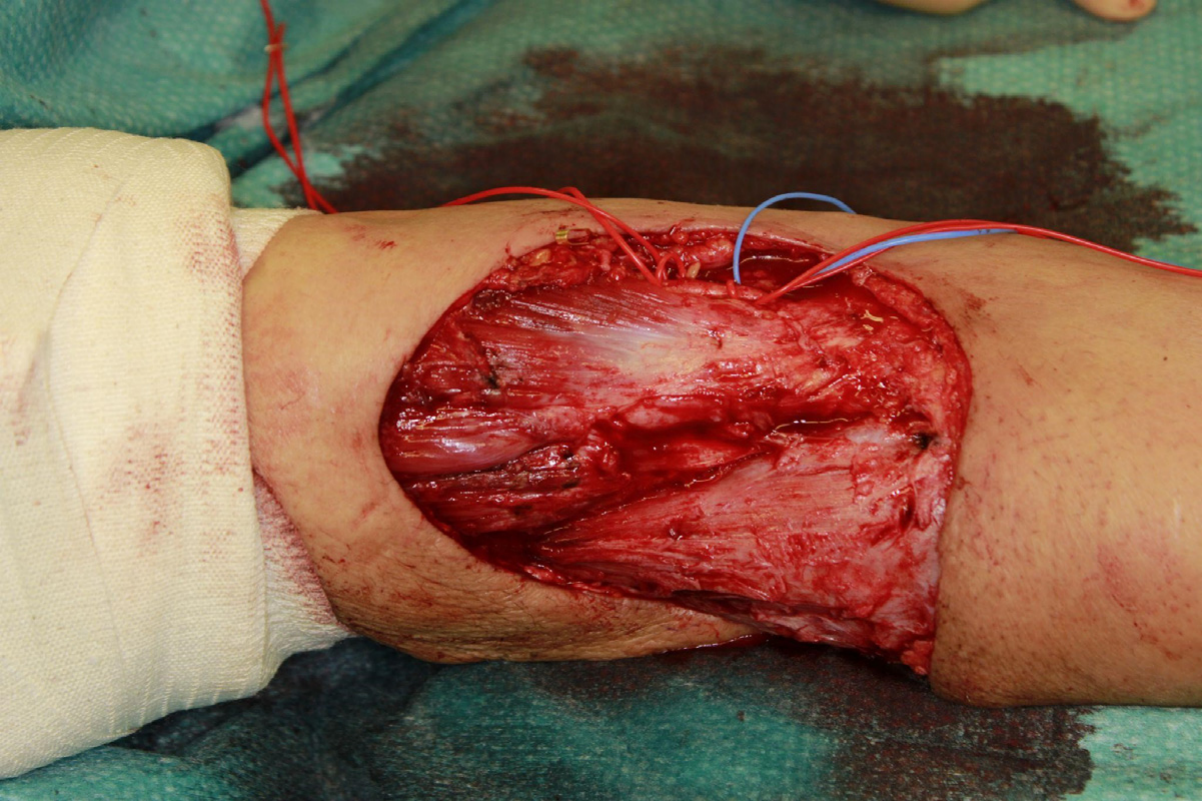
Exemplary extravasation wound after debridement.

After sufficient debridement, the result is shown in Figure 4.

**Figure 5:**
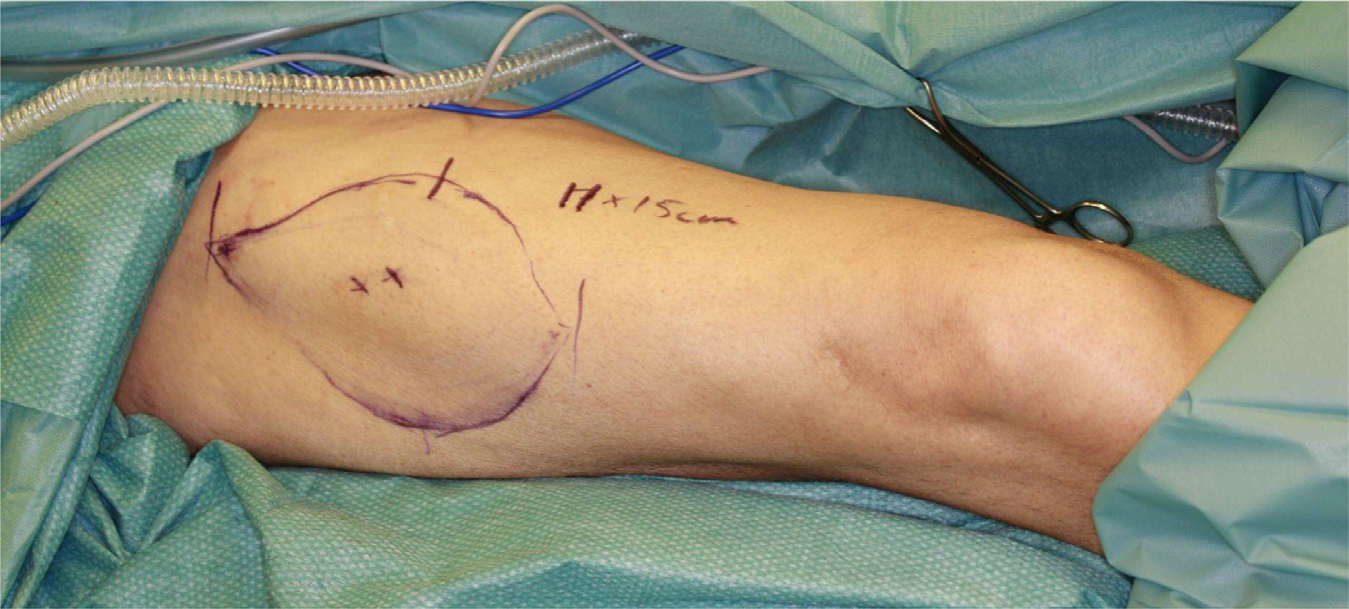
Preoperative markings of ALT flap.

Then, an ALT flap was necessary for coverage of the exposed vessels, nerves and tendons. Preoperative markings are shown in Figure 5.

**Figure 6:**
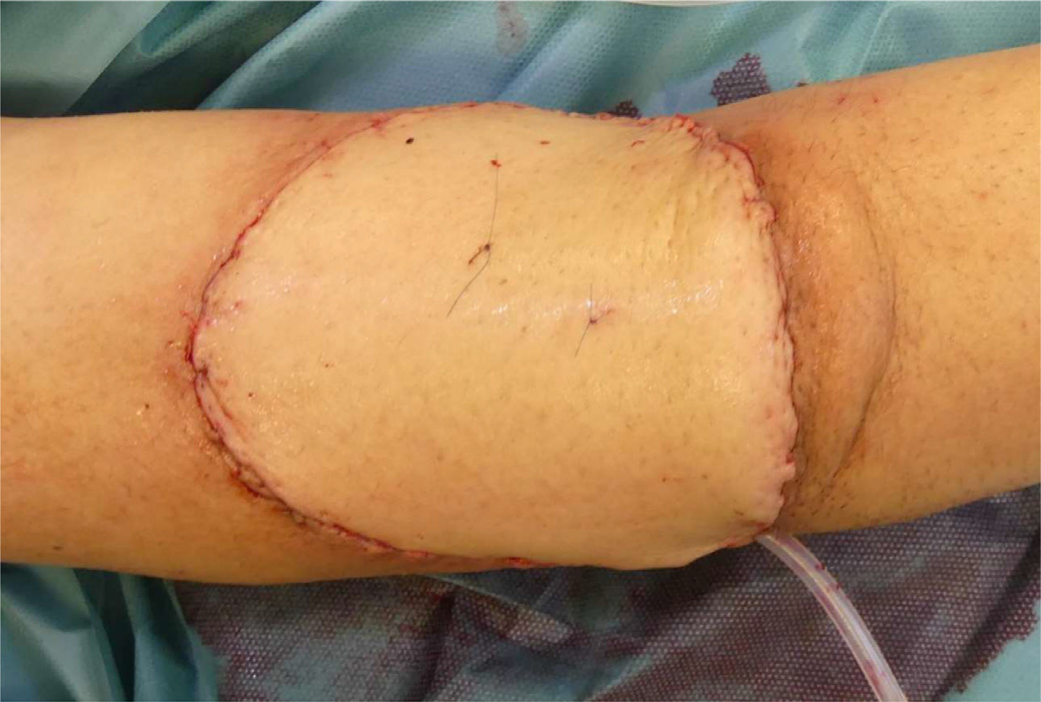
ALT flap directly after the operation.

The flap was harvested and inserted immediately. It survived fully (Figure 6).

**Figure 7:**
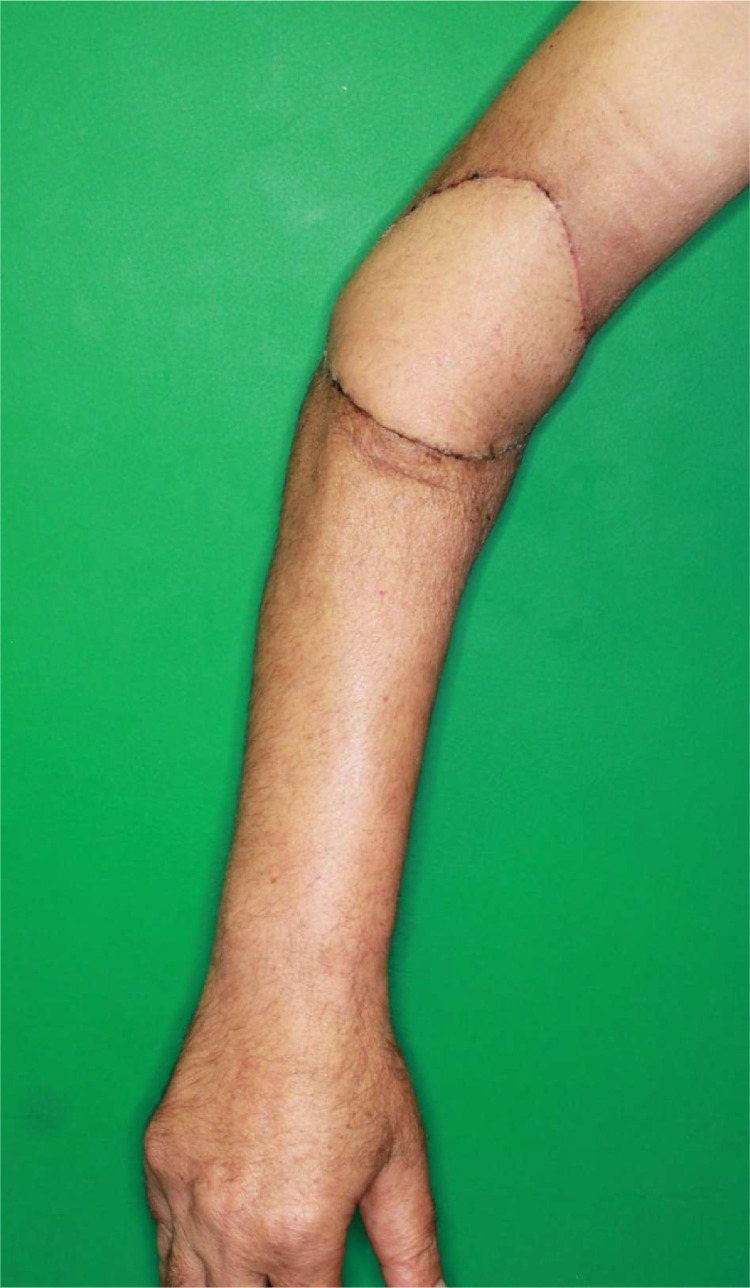
Outcome 2 months post operation.

At 6 months post-injury, the aesthetic and functional outcomes were favorable (Figure 7).

### Complications of extravasation

**Figure 8:**
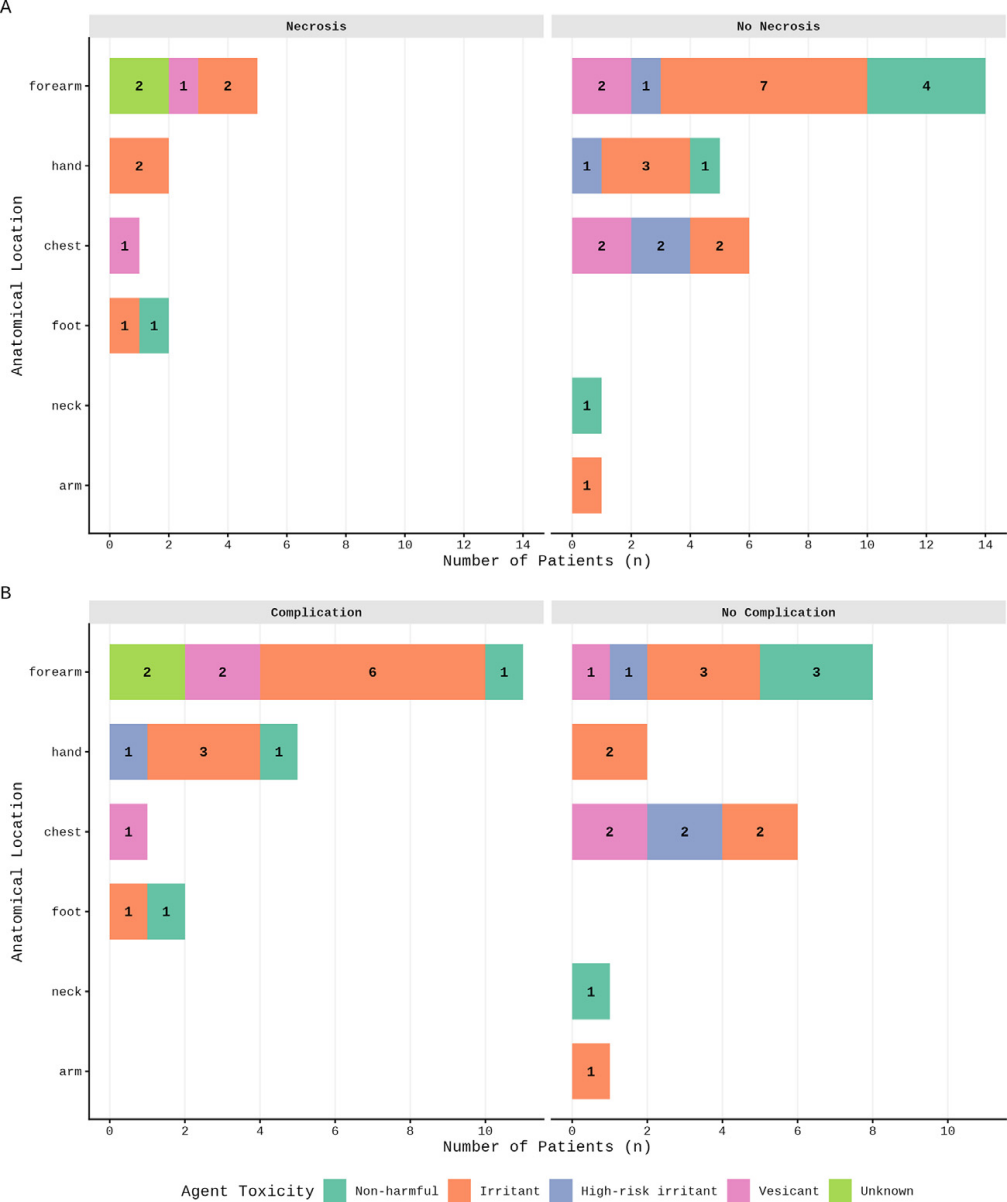
Types of extravasation agents affecting different body parts. Note. (A) Distribution of tissue necrosis across anatomical locations. (B) Distribution of all clinical complications (including necrosis, infection, and compartment syndrome) across anatomical locations. Data is categorized by agent toxicity according to clinical classification. Locations are ordered by total case volume.

Extravasated agents as well as the underlying diagnosis that required the administration of therapeutic or diagnostic agents that led to the extravasation injury differed widely between patients. Overall, 16 of the 37 patients developed a severe complication. The incidence of complications varied significantly across anatomical sites, reflecting the uneven distribution of both the initial injuries and the specific extravasation agents involved (Figure 8).

In our cases of extravasation, skin defects were most frequently observed in acral regions such as the foot and hand. These parts witnessed laceration of their skin without usage of any cytotoxic substance. However, the forearm and chest were more susceptible to chemotherapy administration and thus more likely to show chemotherapy-associated tissue loss.

A broad spectrum of surgical interventions was employed, tailored to the timing of referral and clinical severity. For patients referred promptly without established complications, management focused on preventive measures such as SWOP or simple incision and drainage. In cases of early-onset compartment syndrome, urgent surgical fasciotomy was performed. Conversely, late-stage presentations involving tissue necrosis necessitated extensive debridement and complex flap reconstruction to address significant soft-tissue loss.

**Figure 9:**
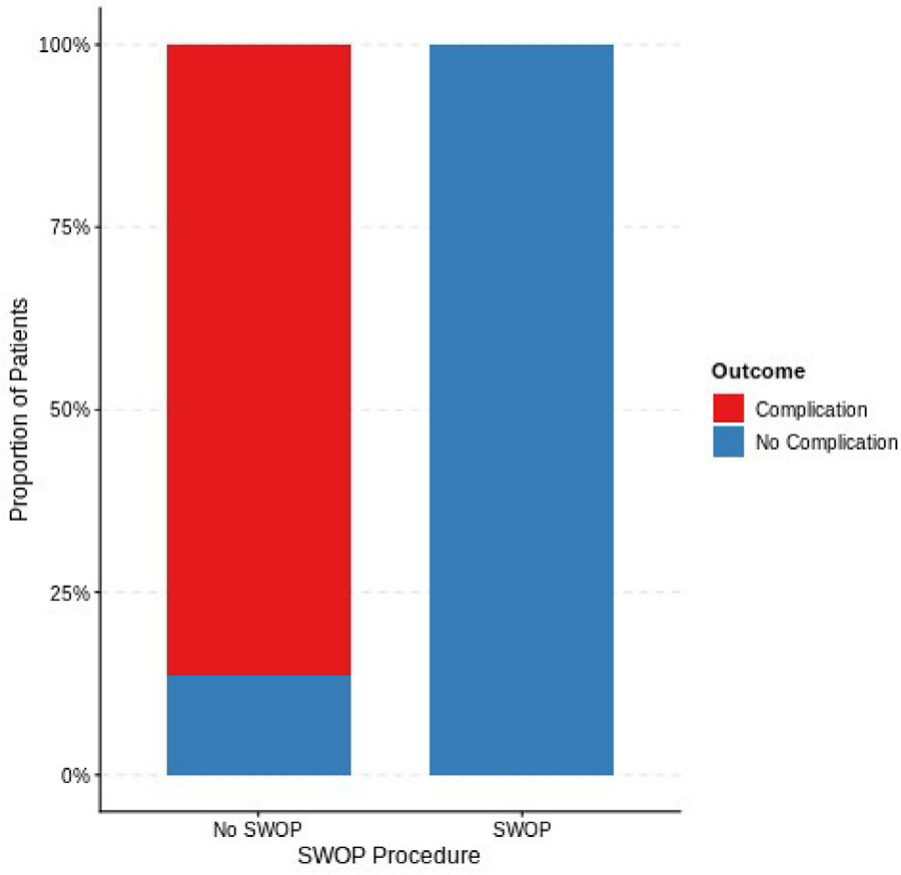
Proportion of Complications by SWOP Status. Note. Proportion of clinical outcomes (complication vs. no complication) following extravasation injury, stratified by the performance of a Subcutaneous Wash-Out Procedure (SWOP). *N* = 37.

Reconstructive complexity (e.g., ALT flaps) was primarily associated with delayed referral rather than failed primary SWOP. Notably, no complications were reported in the subgroup of patients receiving SWOP (Figure 9). The two patients requiring ALT flaps were tertiary referrals who presented to our department with established, deep-tissue necrosis involving tendons and nerves several days post-injury.

### Predictors of extravasation injury evaluated with Bayesian logistic regression

**Figure 10:**
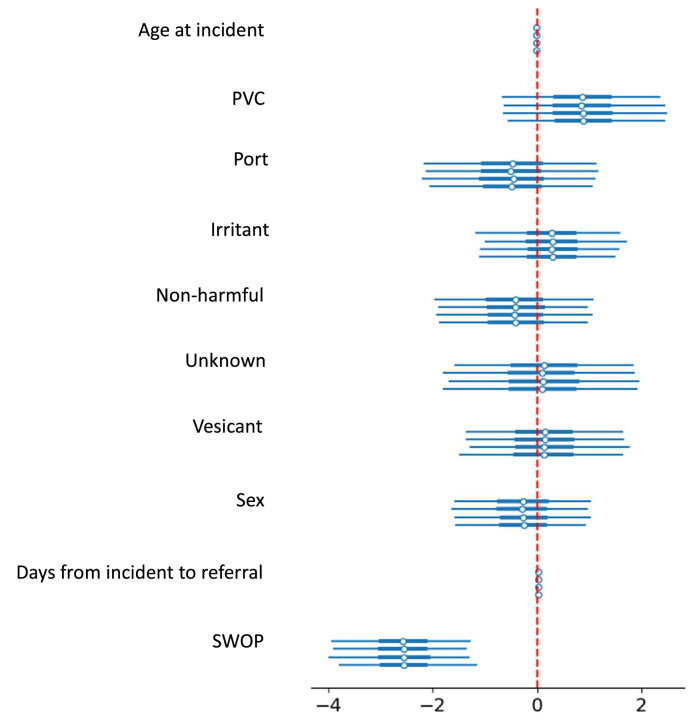
Forest Plot of Bayesian Logistic Regression Coefficients for Extravasation Complications. Note. This forest plot summarizes the Bayesian logistic regression results for factors associated with severe extravasation complications. The horizontal axis represents log-odds coefficients, where dots indicate posterior means and horizontal bars represent 94% High-Density Intervals (HDI). The four vertical bars per variable represent the independent MCMC chains, demonstrating high convergence and model stability. The dashed red line at zero indicates the null effect. The Subcutaneous Wash-Out Procedure (SWOP) is the only statistically credible predictor (Mean: −2.57, HDI: [−3.90, −1.22]). While intervals for referral delay, catheter types, and agent classifications cross zero, their positioning reflects expected clinical trends (e.g., increased risk with delay and peripheral catheters) that support the internal consistency of the dataset.

To identify risk factors associated with severe complications, we employed a Bayesian logistic regression model as the frequentist approach did fail due to almost complete separation.^19^ This approach provided stable estimates for our cohort, with excellent model convergence across all parameters (Figure 10).

The analysis identified SWOP as the primary credible predictor of clinical outcomes. We observed a strong association: the posterior mean coefficient for SWOP was −2.571, with a 94% High-Density Interval (HDI) of [−3.896, −1.215]. Since this interval does not include zero, there is a high degree of certainty that SWOP and severe complications did not coincide within this cohort.

Regarding the route of administration, the model showed that peripheral venous catheters (Mean = 0.881; HDI: [−0.648, 2.457]) and ports (Mean = −0.487; HDI: [−2.128, 1.166]) did not reach the threshold for statistical credibility in this specific multivariable iteration, as their credible intervals overlapped zero.

Similarly, other clinical variables including patient age (Mean = −0.007), biological sex (Mean = −0.269), and the type of agent (e.g., vesicant vs. irritant) did not demonstrate a credible influence on the outcome. Notably, the time from injury to referral (days) showed a positive trend toward increased risk (Mean = 0.022), but its interval (HDI: [−0.004, 0.050]) also contained zero, suggesting that in this surgical cohort, the protective effect of the intervention (SWOP) outweighed the impact of referral delays. While not reaching the threshold for statistical significance, the clinical trends observed—specifically the increased risk associated with peripheral venous catheters (Mean = 0.88) and irritant agents (Mean = 0.29), as well as the positive correlation between referral delay and complications (Mean = 0.02)—align with established clinical expectations. Furthermore, the non-significant protective trend of non-harmful agents (Mean = −0.42) reinforces the internal consistency and credibility of our dataset.

## Discussion

This study provides a detailed analysis of severe extravasation injuries at a tertiary care center that necessitated plastic surgery consultation. In this highly selected cohort, we observed an association between the performance of SWOP and a lower incidence of necrosis. However, this must be interpreted with caution as it does not only show a protective effect of SWOP against severe complications but also correct patient identification as SWOP is not the right treatment for compartment syndrome. Additionally, the association of high risk agents with complications adds to the credibility of our dataset although not significant due to our small sample size.

Although gathered, the volume of extravasated agent remained not more than an estimate, because an exact measurement of the administered fluid was not performed in any case. Additionally, no volumetry was performed and no measurements of skin tension ware assessed other than clinical evaluation. The amount of leakage due to dislocated i.v. catheter or the amount of hemorrhage adding to subcutaneous fluid volume remain unclear.

Infiltration and extravasation are commonly recognized adverse events, particularly among high-risk groups such as newborns in neonatal intensive care units (NICUs), where studies have reported infiltration rates as high as 70%.^22^ Incidence rates vary across the general patient population. For instance, Simin et al.^30^ observed a 16% infiltration rate in more than 1400 intravenous catheters, while Marsh et al.^28^ documented a 32% catheter failure rate that included infiltration cases. Conversely, some healthcare institutions have reported significantly lower figures, with one facility documenting just 14 events over an 18-month span.^23^ It is believed that infiltration may often go under-reported due to the infrequent occurrence of serious or lasting complications. Supporting this, a recent analysis of 495 infiltration events showed that severe outcomes were relatively uncommon - soft tissue infections occurred in 8.6% of cases, necrosis in 3.2%, ulceration in 1.9%, and only 5.1% of patients experienced lasting impairment.^24^ While these findings highlight that most infiltration events are mild, our study intentionally concentrates on the more severe end of the spectrum - cases significant enough to necessitate specialized surgical evaluation.

It is important to note that our cohort consists exclusively of patients whose injuries were severe enough to warrant a plastic surgery referral. Extravasations from CVCs, while potentially catastrophic (e.g., causing mediastinitis or hydrothorax), may be managed primarily by other specialties (e.g., thoracic/head and neck surgery or intensive care) or may be so rapidly fatal that they do not result in a consultation for localized tissue reconstruction. In contrast, PVC and port extravasations occur in the constrained subcutaneous tissues of the limbs and chest wall, respectively. The literature confirms that the vast majority of all extravasation events originate from PVCs, simply due to their widespread use, making their predominance in our sample unsurprising.^25^

Beyond selection and anatomical factors, the technical circumstances of catheter placement and maintenance likely contribute to the observed risk profiles. In clinical practice, peripheral venous access and port-chamber punctures are recurring tasks that may be performed by personnel with varying levels of experience; repeated attempts can compromise vessel integrity and increase the risk of interstitial leakage. In contrast, CVCs in our institution are typically placed by specialized teams using real-time ultrasound guidance, followed by rigorous verification of function before use. Furthermore, the anatomical depth of the delivery site plays a protective role. The tip of a CVC is sequestered within high-flow, large-caliber vessels deep to the muscular fascia, providing a substantial distance between catheter outlet and “soft-tissue buffer” that may mitigate the immediate impact of an extravasation. Conversely, the superficial positioning of PVCs and port reservoirs means that the noxious agent is deposited in immediate proximity to the dermal blood supply, leaving little margin for error before irreversible tissue compromise occurs.

The strong association of SWOP with the absence of clinical complications is a key takeaway.In our cohort, patients who underwent this minimally invasive procedure had drastically lower odds of developing necrosis or compartment syndrome. This aligns with the original intent of the procedure as described by Gault and others, which is to dilute and remove the noxious agent from the subcutaneous tissue before irreversible cytotoxic damage or ischemic pressure can occur.^12^, ^13^, ^27^ Our data provides statistical support for the efficacy of SWOP in a cohort of already severe injuries, suggesting that its early implementation upon recognition of significant extravasation should be a primary consideration to prevent further morbidity and the need for more extensive surgery like debridement and flap reconstruction.

Perhaps the most unexpected result of our multivariable analysis was the lack of a statistically significant association between the type of extravasated agent and the risk of complications. This finding is in direct contrast to a large body of literature and the information presented in our introduction, which firmly establishes vesicant chemotherapies as a primary cause of severe tissue necrosis.^31^^,^^32^ Several factors likely explain this discrepancy. Firstly, our small sample size (*n* = 37) severely limits the statistical power of the model, making it difficult to detect a true effect that may exist. Secondly, the severe selection bias of our cohort may play a role; the injuries caused by other agents in our study (e.g., high-osmolality parenteral nutrition) were, by definition, severe enough to require plastic surgical consultation, potentially creating a “ceiling effect” where the baseline severity of all included cases was high, thus washing out the differential impact of chemotherapy.^33^^,^^34^ Still, the different types of agents show anticipated tendencies (Figure 10), but in a multivariable model confounded by treatment (e.g., SWOP) and catheter type, this signal was lost.

## Limitations

This study has several important limitations. Its retrospective, single-center design inherently limits the generalizability of our findings. Data was reliant on existing medical records, which may contain inaccuracies or omissions. The most significant limitation is the profound selection bias, extravasations that were referred to plastic surgery. This prevents us from calculating the true incidence of complications or identifying risk factors for the initial extravasation event itself. Moreover, extravasations referred to other disciplines are not integrated in our study population and hence limit the accuracy regarding injuries of the neck and thorax. The small sample size restricts the precision of our estimates, as reflected in the wide credible intervals for the odds ratios, and limits our ability to analyze specific extravasated agents or more granular patient factors.

## Conclusion

Our study of severe extravasation injuries highlights critical clinical insights. Our experience suggests that SWOP is a valuable intervention for mitigating the progression to tissue necrosis for mitigating the progression to severe complications, and its use should be strongly encouraged. While extravasations from any catheter can be dangerous, those from PVCs and ports located in the limbs and chest wall represent a high-risk group for the types of complications that necessitate reconstructive surgery. The failure to identify chemotherapy as a risk factor in our model should not be misinterpreted as evidence of its safety; rather, it underscores the severe nature of injuries that can be caused by a variety of non-chemotherapeutic agents, it hints at a potentially higher awareness and faster treatment of extravasation of chemotherapy compared to non-therapeutic agents and it highlights the methodological limitations of a small, highly selected cohort. Future research should focus on prospective, multi-center registries to capture all extravasation events, which would allow for a more robust and unbiased identification of risk factors for both the initial event and its progression to severe, debilitating injury.

## Funding

None.

## Ethical approval

This study was conducted in accordance with the principles of the Declaration of Helsinki. As a retrospective analysis conducted at a University Hospital, the study utilized anonymized data under the institution’s broad consent policy; consequently, formal approval from the local Ethics Committee was waived. All data were processed and anonymized by the hospital’s data protection institute prior to analysis. Notwithstanding the general waiver for aggregate data, specific written informed consent was obtained from the patient featured in the case presentation for the usage and publication of clinical photography, in accordance with editorial guidelines.

## Declaration of competing interest

None declared.

## References

[1] Definition of extravasation - NCI dictionary of cancer terms - NCI (2011). https://www.cancer.gov/publications/dictionaries/cancer-terms/def/extravasation.

[2] Goolsby T.V., Lombardo F.A. (2006). Extravasation of chemotherapeutic agents: prevention and treatment. Semin Oncol.

[3] Loubani O.M., Green R.S. (2015). A systematic review of extravasation and local tissue injury from administration of vasopressors through peripheral intravenous catheters and central venous catheters. J Crit Care.

[4] Reeves D. (2007). Management of anthracycline extravasation injuries. Ann Pharmacother.

[5] Jordan K., Behlendorf T., Mueller F., Schmoll H.J. (2009). Anthracycline extravasation injuries: management with dexrazoxane. Ther Clin Risk Manag.

[6] De Wit M., Ortner P., Lipp H.P. (2013). Management of cytotoxic extravasation - ASORS expert opinion for diagnosis, prevention and treatment. Oncol Res Treat.

[7] Pérez Fidalgo J.A., García Fabregat L., Cervantes A., Margulies A., Vidall C., Roila F. (2012). Management of chemotherapy extravasation: ESMO–EONS Clinical Practice Guidelines. Ann Oncol.

[8] Ener R.A., Meglathery S.B., Styler M. (2004). Extravasation of systemic hemato-oncological therapies. Ann Oncol.

[9] Pluschnig U., Haslik W., Bayer G. (2015). Outcome of chemotherapy extravasation in a large patient series using a standardised management protocol. Support Care Cancer.

[10] Boulanger J., Ducharme A., Dufour A., Fortier S., Almanric K. (2015). Management of the extravasation of anti-neoplastic agents. Support Care Cancer.

[11] Onesti M.G., Carella S., Fioramonti P., Scuderi N. (2017). Chemotherapy extravasation management: 21-year experience. Ann Plast Surg.

[12] Gault D.T. (1993). Extravasation injuries. Br J Plast Surg.

[13] Steiert A., Hille U., Burke W. (2011). Subcutaneous wash-out procedure (SWOP) for the treatment of chemotherapeutic extravasations. J Plast Reconstr Aesthet Surg.

[14] Chfiri A., Karti S., Jalal A. (2022). Extravasation: surgical management and prevention. Eur J Med Health N Hav Sci.

[15] Langstein H.N., Duman H., Seelig D., Butler C.E., Evans G.R.D. (2002). Retrospective study of the management of chemotherapeutic extravasation injury. Ann Plast Surg.

[16] von Elm E., Altman D.G., Egger M., Pocock S.J., Gøtzsche P.C., Vandenbroucke J.P. (2007). The strengthening the reporting of observational studies in epidemiology (STROBE) statement: guidelines for reporting observational studies. Lancet.

[17] Wickham H. (2016).

[18] Kaas-Hansen B.S. (2024). epiben/humap. https://github.com/epiben/humapr.

[19] Kimball A.E., Shantz K., Eager C., Roy J. (2019). Confronting quasi-separation in logistic mixed effects for linguistic data: a Bayesian approach. J Quant Linguist.

[20] R Core Team (2024).

[21] Stan Development Team. RStan: the R interface to Stan [Internet]. 2025. R package version 2.32.7. Available from: https://mc-stan.org/.

[22] Sangam S.L. (2019). Quality improvement measures for early detection of severe intravenous infiltration in infants. BMJ Open Qual.

[23] Tewfik G. (2020). Under-reporting of a critical perioperative adverse event: intravenous infiltration and extravasation. Drug Healthc Patient Saf.

[24] Gibian J.T., Zakria D., March C., Schaheen B., Drolet B.C. (2022). Outcomes and management of peripheral intravenous infiltration injuries. HAND.

[25] Dougherty L. (2008). IV therapy: recognizing the differences between infiltration and extravasation. Br J Nurs Mark Allen Publ.

[26] Deutsche Krebsgesellschaft (DKG), Deutsche Gesellschaft für Hämatologie und Medizinische Onkologie (DGHO), Deutsche Gesellschaft für Radioonkologie (DEGRO). S3-Leitlinie Supportive Therapie bei onkologischen PatientInnen. 2025 [cited 2025 July 15]. Available from: https://register.awmf.org/de/leitlinien/detail/032-054OL

[27] Giunta R., Akpaloo J., Kovacs L., Biemer E. (2002). Technik der subkutanen Spülung bei hochtoxischen Paravasaten - Ein Kurzbeitrag. Handchir · Mikrochir · Plast Chir..

[28] Marsh N., Webster J., Larsen E., Cooke M., Mihala G., Rickard C.M. (2018). Observational study of peripheral intravenous catheter outcomes in adult hospitalized patients: a multivariable analysis of peripheral intravenous catheter failure. J Hosp Med.

[29] McCullen K.L., Pieper B. (2006). A retrospective chart review of risk factors for extravasation among neonates receiving peripheral intravascular fluids. J Wound Ostomy Continence Nurs.

[30] Simin D., Milutinović D., Turkulov V., Brkić S. (2019). Incidence, severity and risk factors of peripheral intravenous cannula-induced complications: an observational prospective study. J Clin Nurs.

[31] Kreidieh F.Y. (2016). Overview, prevention and management of chemotherapy extravasation. World J Clin Oncol.

[32] Schulmeister L. (2011). Extravasation management: clinical update. Semin Oncol Nurs.

[33] Reynolds P.M., MacLaren R., Mueller S.W., Fish D.N., Kiser T.H. (2014). Management of extravasation injuries: a focused evaluation of noncytotoxic medications. Pharmacotherapy.

[34] MacCara M.E. (1983). Extravasation: a hazard of intravenous therapy. Drug Intell Clin Pharm.

